# Fruit and vegetable leaf disease recognition based on a novel custom convolutional neural network and shallow classifier

**DOI:** 10.3389/fpls.2024.1469685

**Published:** 2024-09-30

**Authors:** Syeda Aimal Fatima Naqvi, Muhammad Attique Khan, Ameer Hamza, Shrooq Alsenan, Meshal Alharbi, Sokea Teng, Yunyoung Nam

**Affiliations:** ^1^ Department of Computer Science, HITEC University, Taxila, Pakistan; ^2^ Department of Artificial Intelligence, College of Computer Engineering and Science, Prince Mohammad Bin Fahd University, Al Khobar, Saudi Arabia; ^3^ Information Systems Department, College of Computer and Information Sciences, Princess Nourah bint Abdulrahman University, Riyadh, Saudi Arabia; ^4^ Department of Computer Science, College of Computer Engineering and Sciences, Prince Sattam Bin Abdulaziz University, Alkharj, Saudi Arabia; ^5^ Department of ICT Convergence, Soonchunhyang University, Asan, Republic of Korea

**Keywords:** cucumber crop, apple fruit, deep learning, information fusion, optimization, explainable deep learning, shallow classifier

## Abstract

Fruits and vegetables are among the most nutrient-dense cash crops worldwide. Diagnosing diseases in fruits and vegetables is a key challenge in maintaining agricultural products. Due to the similarity in disease colour, texture, and shape, it is difficult to recognize manually. Also, this process is time-consuming and requires an expert person. We proposed a novel deep learning and optimization framework for apple and cucumber leaf disease classification to consider the above challenges. In the proposed framework, a hybrid contrast enhancement technique is proposed based on the Bi-LSTM and Haze reduction to highlight the diseased part in the image. After that, two custom models named Bottleneck Residual with Self-Attention (BRwSA) and Inverted Bottleneck Residual with Self-Attention (IBRwSA) are proposed and trained on the selected datasets. After the training, testing images are employed, and deep features are extracted from the self-attention layer. Deep extracted features are fused using a concatenation approach that is further optimized in the next step using an improved human learning optimization algorithm. The purpose of this algorithm was to improve the classification accuracy and reduce the testing time. The selected features are finally classified using a shallow wide neural network (SWNN) classifier. In addition to that, both trained models are interpreted using an explainable AI technique such as LIME. Based on this approach, it is easy to interpret the inside strength of both models for apple and cucumber leaf disease classification and identification. A detailed experimental process was conducted on both datasets, Apple and Cucumber. On both datasets, the proposed framework obtained an accuracy of 94.8% and 94.9%, respectively. A comparison was also conducted using a few state-of-the-art techniques, and the proposed framework showed improved performance.

## 1 Introduction

Identifying plant diseases has been a major problem in the agricultural sector in recent years (Tekkeşin, 2019). It is critical to accurately diagnose and recognize the disease at early stages (Rumpf et al., 2010; Gavhale and Gawande, 2014). Unnecessary substantial economic losses can be avoided due to the early detection of diseases in agricultural production (Savary et al., 2019). The quantity and quality of agricultural products are greatly affected by diseased crops, which destroy the natural condition of the crop by altering and stopping critical activities, including transpiration, germination, pollination, fertilization, and photosynthesis (Wang X. et al., 2015). Moreover, plant disease symptoms typically manifest as visual abnormalities on leaves at a certain development stage. Therefore, using machine learning algorithms to evaluate plant and crop leaf images makes it feasible to identify leaf illnesses automatically (Ngugi et al., 2021; Haridasan et al., 2023; Ngongoma et al., 2023).

The manual diagnosis of leaf disease is a difficult and hectic process (Ngugi et al., 2021). In addition, an expert is required, which is not an easy task (Haridasan et al., 2023). Therefore, agriculture’s computerized technique is widely needed to diagnose and classify diseases in leaf images at the early stages Early diagnosis not only improves food quality but also increases food quantity, which can benefit the national economy (Ngongoma et al., 2023). The proficiency to identify plant disease at an early stage allows us to diagnose and eradicate infectious diseases in plants before apparent symptoms arise, reducing the massive economic losses that would otherwise occur and leading the ton of food to be safeguarded from the impending outbreak (Sharon et al., 2010). The traditional computerized techniques are usually based on machine learning models such as support vector machine (SVM) (Bonkra et al., 2024) and decision trees (DT) (Sajitha et al., 2024). These models accept input as a feature vector extracted through a handcrafted approach, such as shape, color, and texture (Al-Hiary et al., 2011; Gulhane and Gurjar, 2011; Patil and Kumar, 2011). In several works, feature selection techniques are introduced to select the best features for the classification and reduce the computational time. However, it isn’t easy when a large dimensional vector is passed as an input (Amiriebrahimabadi et al., 2024). There are a few well-known feature selection techniques, such as principle component analysis (PCA), Genetic Algorithm (GA) (Khan et al., 2019), particle swarm optimization (PSO), and a few more (Jain and Dharavath, 2023; Jena et al., 2024; Vijay and Pushpalatha, 2024).

The more recent development in artificial intelligence is deep learning (DL), employed for disease detection and classification (Nawaz et al., 2024). Convolutional neural network (CNN) is a specialized type of deep learning (DL) that is utilized to extract the features of an object or image from several hidden layers (Joseph et al., 2024). Recently, many techniques have been introduced for detecting and classifying plant diseases based on transfer learning (TL) and custom CNN. Several pre-trained models were opted for in the TL phase, and deep features were extracted (Ritharson et al., 2024). In a few techniques, models are trained from scratch due to the complex nature of selected datasets (Jha et al., 2024). A few well-known pre-trained models that are used in the literature for plant diseases are AlexNet (Krizhevsky et al., 2017), VGG16 and Vgg19 (Simonyan and Zisserman, 2014), ResNet (He et al., 2016), and EfficientNet (Tan and Le, 2019). These models work better for the balanced and easy nature of plant datasets; however, for complex, imbalanced, and small datasets, these pre-trained models do not perform well (Ganatra and Patel, 2021). Therefore, a custom model can be designed based on the literature review knowledge and the number of learnable. There are a few recent works that used deep learning architectures for the effective classification of plant diseases (Saleem et al., 2019; Duong et al., 2020).

Several deep-learning techniques have been introduced in the literature to classify plant diseases (Pradhan et al., 2024; Xu and Zhang, 2024). Recent works have been based on pre-trained networks and the fusion of different networks (Ma et al., 2018). Fang et al (2024). presented a lightweight bilinear CNN architecture for apple leaf disease detection and classification. The focus was on the small infected regions of the apple leaf images. For this purpose, the presented CNN architecture consists of two subnetworks. They used the bilinear concat function for feature extraction, which was further employed for classification through classification techniques. The presented method obtained improved accuracy than the unimproved LeNet-5. Haiping et al (Si et al., 2024). presented a dual-brach model for apple leaf disease classification. The presented model integrates two separate networks, CNN and Swin Transformer. The purpose of CNN in this work is to extract the local information, whereas the global information is computed through the Swin Transformer. In addition, the information of these models is fused using a fusion module based on the residual, sqeeze, and excitation mechanisms. The experimental process of the presented model is performed on publically available dataset and obtianed recall rate of 97.33% that is improved than the recent methods. Wang et al (Wang et al., 2021). proposed a two-stage recognition model for cucumber leaf diseases. The proposed model was based on U-Net and DeepLabV3+ that later passed to the severity recognition module. The presented method obtaiend the classification accuracy of 92.85% that is improved than the recent works. There are several more recent works that performed classification of plant diseases using deep learning techniques. Saleem et al (Saleem et al., 2020). presented a plant leaf disease detection and classification framework based on TensorFlow and custom deep learning architecture. The presented model is tested on real-time acquired data and obtained an accuracy of 73.07%. Chowdhury et al (2021). described an EfficientNet architecture based on the better performance. They considered EfficientNet-B7 for the classification and obtained an accuracy of 99.89%. Ahmed et al (Ahmed and Reddy, 2021). presented a CNN architecture for diagnosing plant diseases and obtained an accuracy of 94%. Harakannanavar et al (2022). employed machine learning and image processing to identify leaf diseases in tomato plants, achieving high accuracy rates of 88% for SVM, 97% for K-NN, and 99.6% for CNN on disease samples. Jadhav et al (2021). presented a framework for soybean disease identification methods using pre-trained models such as AlexNet and GoogleNet. On these models, they achieved an accuracy rate of 98.75 and 96.25%, respectively. Abbas et al (2021). employed a pre-trained DenseNet121 deep-learning architecture to detect tomato diseases and obtained an average accuracy of 97.11% on the Plant Village dataset. Several Deep-learning techniques have been used to classify plant diseases, with recent works focusing on pre-trained networks and the fusion of different networks. Recent works have improved accuracy rates but all these methods are based on fine-tuning the previously trained models, modifying them, or fusing them to make improvements in the results while they do improve the results they have a major limitation of high parameters and increased computation time.

**Figure 1:**
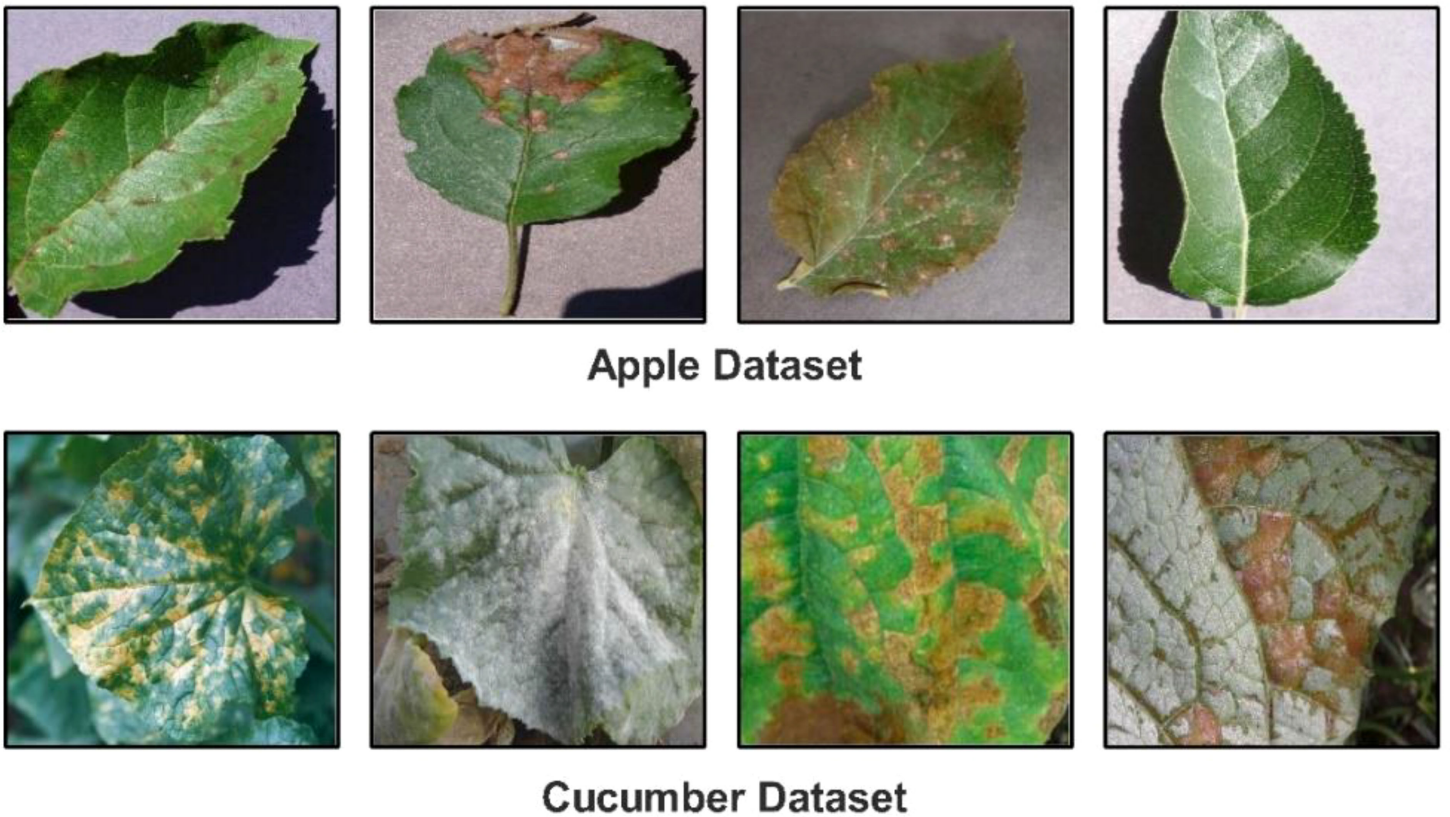
Sample images of selected datasets.

**Problem Statement:** In this work, we considered the following major challenges that impact the performance of the proposed method for Apple and Cucumber leaf disease recognition. The major challenges are as follows: i) low contrast disease symptoms are not accurately considered in the deep learning models for the features extraction that, in return, classify as healthy regions; ii) pre-trained models have a large number of parameters such as VGG16 and VGG19 models total learnable is above 140 million; hence, models that have higher number of learnable consumed more time in training and reduced the correct precision rate; iii) fusion of features from the impact of the different sources on the classification accuracy (false positive rate) due to redundant and irrelevant information. Hence, proposing an efficient solution that consumes minimum resources and returns improved accuracy and precision rate is important. Our major contributions to this work are as follows:

▪ We proposed a novel Custom CNN architecture with a shallow neural network and explainable AI (XAI) for the classification of Cucumber (powdery mildew, anthracnose, blight, downy mild, and angular leaf spot) and apple leaf diseases (Apple Scab, Apple Cedar Rust, Black Rot and healthy). **Figure 1** shows the disease images.

▪ A hybrid disease contrast enhancement technique is proposed based on the Bi-LSTM and Haze reduction for the better feature learning.

▪ We proposed two custom models named lightweight Bottleneck Residual with Self-Attention (BRwSA) and Inverted Bottleneck Residual with Self-Attention (IBRwSA) in order to increase the precision rate.

▪ Features are extracted from the self-attention layer and fused using a concatenation formula later optimized using an improved human learning optimization algorithm.

## 2 Proposed methodology

**Figure 2:**
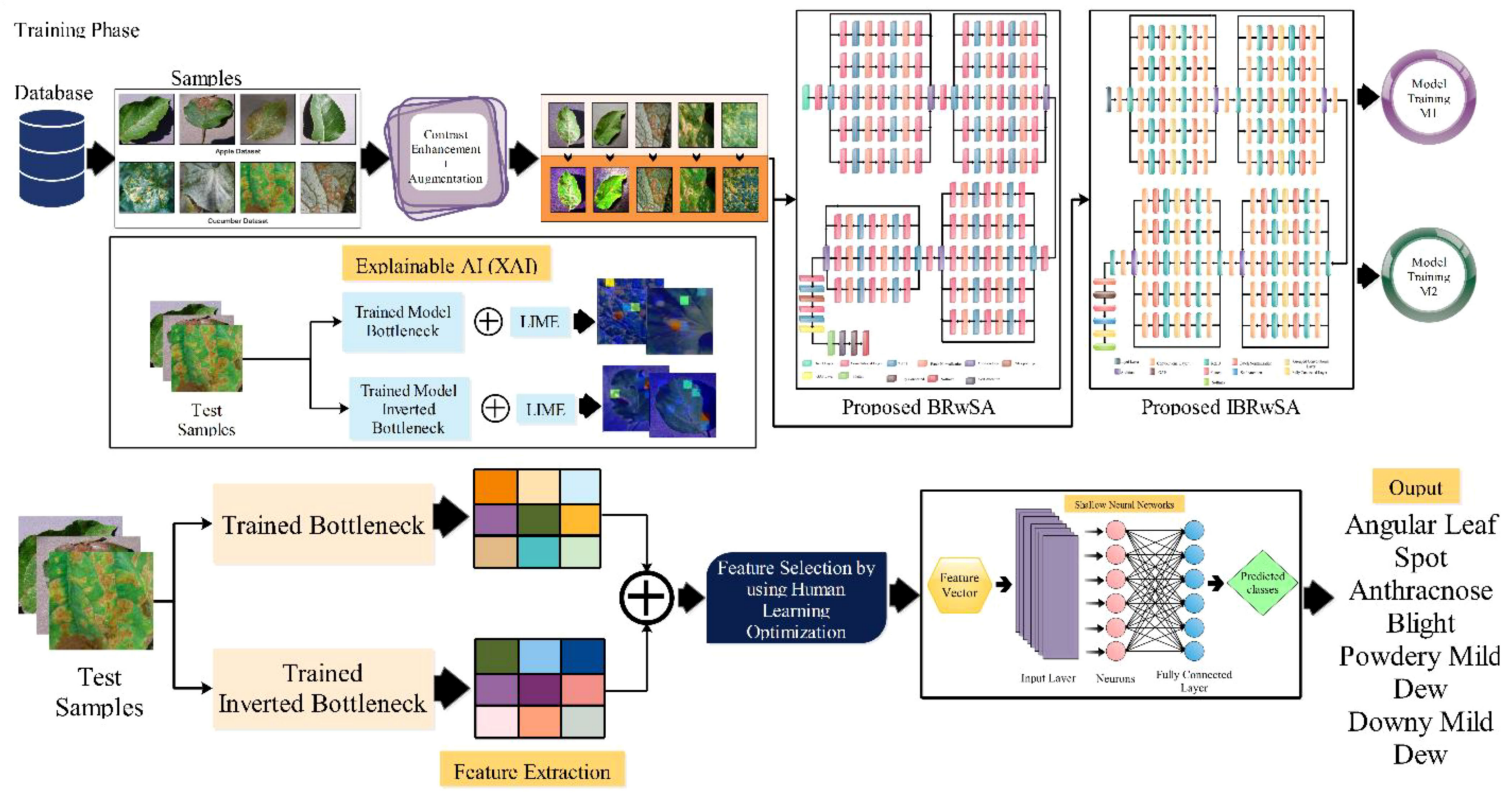
Proposed architecture of apple and cucumber leaf disease recognition.

The proposed methodology of the presented work is discussed in this section with detailed mathematical formulation, theoretical aspects, and visual graphs. A hybrid disease contrast enhancement technique is proposed based on the Bi-LSTM and Haze reduction techniques at the initial stage. After that, we proposed two custom models named Bottleneck Residual with Self-Attention (BRwSA) and Inverted Bottleneck Residual with Self-Attention (IBRwSA) to extract deep learning features. Deep features are extracted from the self-attention layer from both models and fused using a concatenation approach. The concatenation vector is optimized in the next step using an improved human learning optimization algorithm that is finally classified using a shallow wide neural network (SWNN) classifier. In addition to that, both trained models are interpreted using an explainable AI technique such as LIME. Based on this approach, it is easy to interpret the inside strength of both models for apple and cucumber leaf disease classification and identification. **Figure 2** shows the detailed architecture of proposed apple and cucumber leaf disease recognition.

### 2.1 Datasets

In this work, we utilized two datasets for the classification of apple and cucumber leaf disease recognition. For apple leaf disease recognition, we utilized the Plant Village dataset (Mohameth et al., 2020); however, for cucumber disease, a private dataset has been employed (Zhang et al., 2017). We only included apple leaf classes from the 39 fruits and vegetables in the Plant Village dataset. The nature of each image of this dataset is RGB, and the total number of images (apple disease) is 3,171. Four classes were included in this dataset: Apple Scab, Apple Cedar Rust, Black Rot, and Healthy (sample images can be seen in **Figure 1**).

There are 407 total images in the Cucumber collection, all of which have RGB nature. This dataset has five classes: powdery mildew, anthracnose, blight, downy mild, and angular leaf spot (sample images can be seen in **Figure 1**). A summary of images in each class is presented in **Table 1**. This table shows that the images are not enough for training purposes; therefore, a data augmentation process is essential.

**Table 1:** Summary of datasets employed for the validation of the proposed architecture.

Apple Dataset		
Class name	No. of Images	After Augmentation
Apple_Healthy	1645	1000
Apple_Cedar Rust	275	1000
Apple_Black Rot	621	1000
Apple_Scab	630	1000
Cucumber Dataset		
Class Name	No. of Images	After Augmentation
Angular_Leaf_Spot	64	1000
Anthracnose	93	1000
Blight	66	1000
Downy_Mildew	97	1000
Powdery_Mildew	87	1000

### 2.2 Contrast enhancement and datasets augmentation

#### 2.2.1 Enhancement

Enhancing the quality of original images by adjusting the intensity value, adding missing information, and rearranging the data more effectively and organized is known as enhancement. The primary advantage of enhancing datasets is increased accuracy. In the case of images, our major aim is to increase the contrast of the disease region and make it visually more apparent. In this work, we proposed a hybrid contrast enhancement technique based on the haze reduction and Bi-histogram equalization techniques. A noise is included and removed in the selected datasets through the haze reduction technique, whereas Bi-Histogram Equalization improves the contrast. Mathematically, this process is defined as follows:

The following mathematical equation can represent the hazed image:


(1) I(X) = J(X)t(X) + L(1−t(X))

Where the observed intensity is  I , the scene of radiance is denoted with J , atmospheric light is defined by L , and a transmission map  t describes the portion of light that reaches the camera. Hence, to recover the scene of radiance J from an estimation of transmission map and atmospheric light, the dazed algorithm is used, which is defined as follows:


(2) J(X) = (I(X)−a)/(max(T(X),T0)) + a   

**Figure 3:**
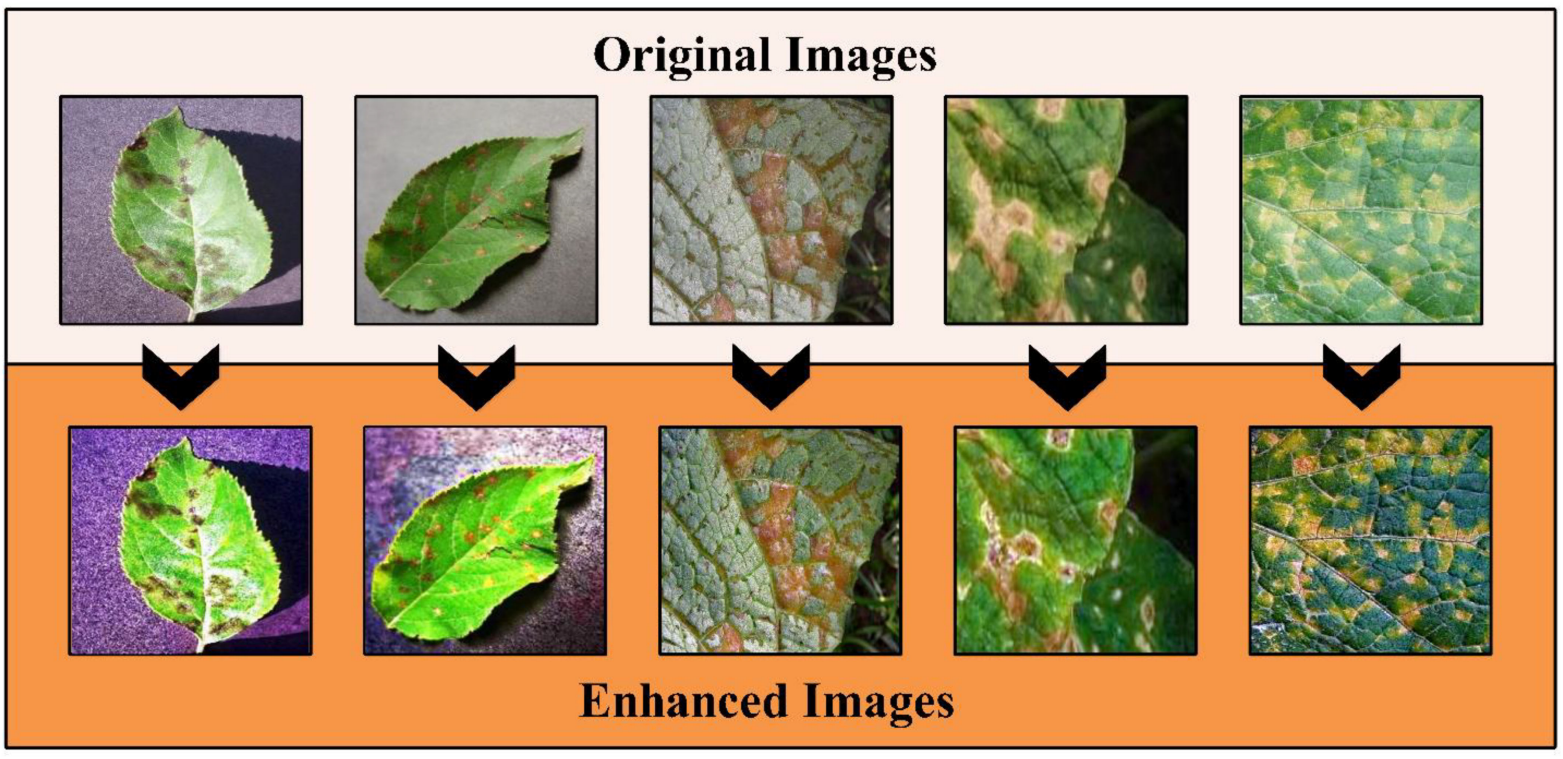
Sample contrast-enhanced images using a hybrid approach.

This technique followed the five steps that started from atmospheric light *L* using a dark channel before restoring the image and performing optional contrast enhancement. The *a* denotes an static parameter that value is 0.2. More information on this method can be read from this work (Park et al., 2014). The output image of this method is passed to Bi-Histogram Equalization (BiHE) to further increase the contrast of the infected regions. The BiHE method is based on five steps such as i) Gaussian filter smoothing of the histogram; ii) Using this smoothed histogram to find local maximums; iii) Designate and map each component to a sophisticated dynamic range; iv) Equalize each histogram independently, and v) Normalization of image brightness. More details on the mathematical form of this method can be seen here (Tang and Isa, 2017). A few sample images after the hybrid contrast enhancement technique are shown in **Figure 3**. In this figure, it is observed that the results enhanced images are clearer than the original images. The resultant enhanced images are later employed for the augmentation process. Like the traditional geometric transformation methods in recent studies, we consider the auto-encoder for generating new images (Kingma and Welling, 2013).

Using this technique, we generated 1000 images of each class. In the generation of images, each image is passed minimum 2 times and few of the images iterated in 3 times. A summary of generated images is presented in **Table 1**.

### 2.3 Proposed bottleneck residual self-attention CNN

Two new deep-learning architectures have been designed in this work for the deep feature extraction of apple and cucumber leaf disease recognition. The pre-trained deep learning models gained a lot of knowledge but did not return enough accuracy (Aggarwal et al., 2023). Therefore, we designed two architectures: Bottleneck Residual with Self-Attention (BRwSA) and Inverted Bottleneck Residual with Self-Attention (IBRwSA).

**Figure 4:**
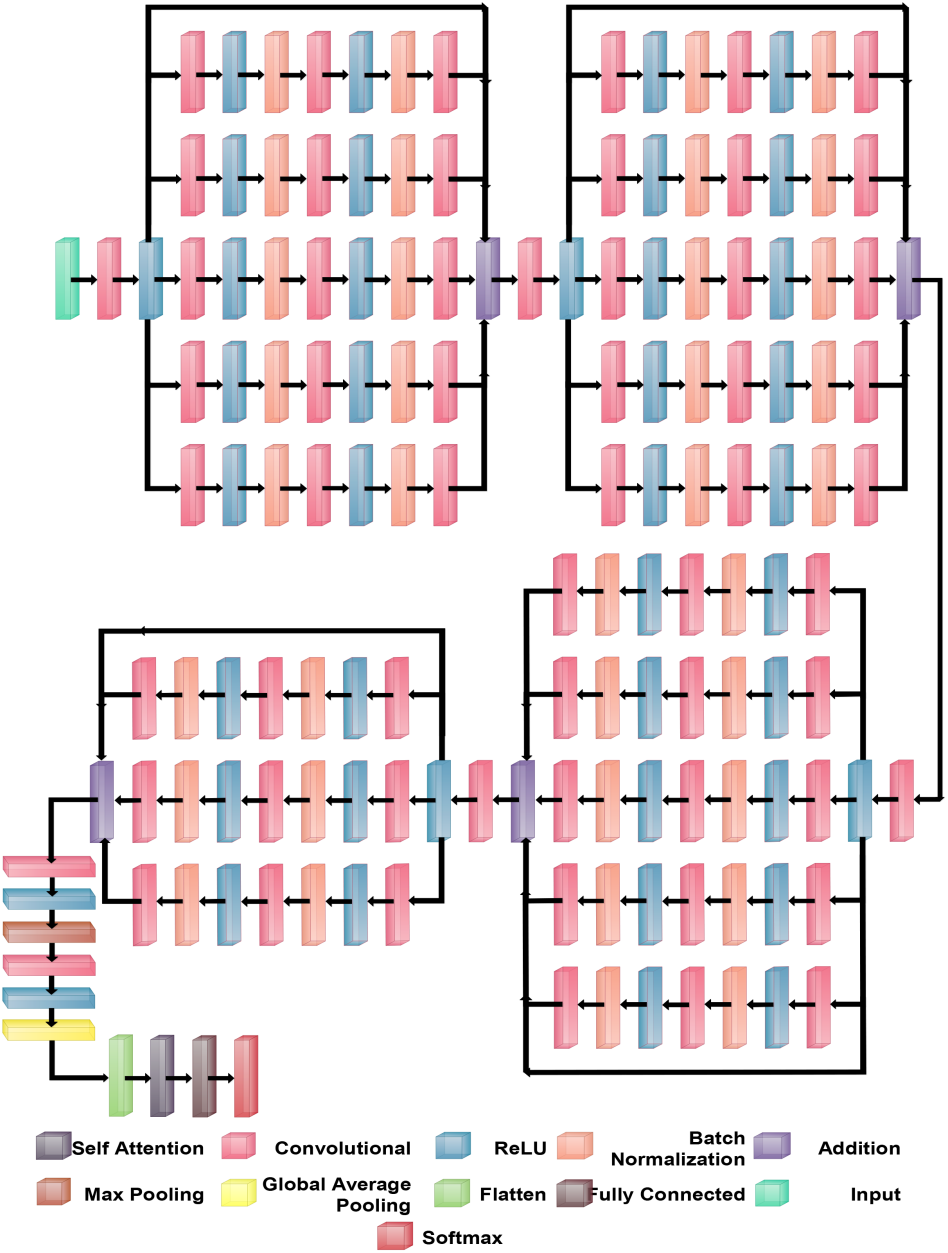
Proposed Bottleneck Residual with Self-Attention (BRwSA) architecture.

BRwSA model consists of a bottleneck residual mechanism whose main objective is to reduce the dimensionality of the feature map while preserving important data, perhaps leading to more efficient and less computationally expensive models. A bottleneck block is a specific type of neural network building block. Each residual function is represented by a stack of three levels- 1×1, 3×3, and 1×1 convolutions. Dimensions are decreased and subsequently increased (restored) by the 1×1 layer. This layer is like a little filter, only examining a small amount of the input data. It makes use of tiny filters with a 1×1 pixel size. The 3×3 convolutional layer uses the larger 3×3 filters to identify complex patterns and features in the data. It operates using the fewer channels produced by the previous 1×1 filter. The third 1×1 convolutional layer performs a second round of 1×1 convolution following the 3×3 convolution. This extra step contributes to the data representation by increasing its feature count, making it more appealing and richer. **Figure 4** illustrates the proposed BRwSA architecture. This figure shows that four blocks are added, and in each block, several layers are added in a parallel fashion using a bottleneck sequence.

#### 2.3.1 Block 1

The first block in this figure consists of five paths, and each path follows the sequence of several layers, such as convolutional, ReLu, batch normalization, max pooling, and addition layer. In addition, a skip connection is also added to overcome the problem of overfitting. The input layer of this model accepts images of dimensions 224×224×3, followed by a convolutional layer of depth size 32, stride of 2×2, filter size of 3×3, and ReLU activation layer.

Subsequently, five bottleneck blocks—consisting of a 32-depth convolutional layer, a 1-by-1 filter with a stride of one, an activation layer for ReLU, and a batch normalization layer—are added in parallel. After that, the 128-depth convolutional layer with a 3×3 filter size, an activation layer of ReLU, and a batch normalization layer comprise the bottleneck structure’s second section. In the last part, a convolutional layer with a depth size of 32 and a filter size of 1×1 has been added. The additional layer is added to connect these parallel blocks with another set of layers. Then, a convolutional layer was added with a ReLU activation layer with a depth size of 64, filter size of 3×3 and stride 2.

#### 2.3.2 Block 2

After that, five parallel residual blocks have been added following the bottleneck pattern. In the first block, a convolutional layer was inserted with a depth size of 128, filter size of 1×1, and stride value of 1, followed by a ReLU activation layer. Then, a batch normalization layer was added. Again, a convolutional layer with a depth size of 256, filter size of 3×3, and stride of 1 with ReLU activation has been added. After this, another batch normalization layer was added to the architecture. Next comes the attachment of the last part of the block, which comprises a 64-depth convolution layer with a 1×1 filter size and one stride. These five bottleneck blocks are concatenated together using an addition layer and a skip connection. After this block, a convolutional layer of depth size 128, stride value of two.

#### 2.3.3 Block 3

The subsequent residual block follows the same structure as the preceding block, which had five bottleneck paths added in parallel, each consisting of 256, 512, and 128 as depth for the convolution layer in the bottleneck structure. The filter size of each block is 1×1, 3×3, and 1×1, respectively. An addition layer concatenates all five blocks of the bottleneck. Later, a convolution layer of 256 depth size, 3×3 filter size, and 2×2 stride with a ReLU activation layer is inserted.

#### 2.3.4 Block 4

In the fourth block, three bottleneck residual paths have been included along with a skip connection. Each bottleneck block has a 512-depth convolution layer with a filter size of 1×1, a stride of 1×1, a ReLU activation layer, and a batch normalization layer. The same depth is opted for the second convolutional layer; however, the filter size of 3×3 has been opted. The last convolutional layer has a 256-depth size, 1×1 filter size, and one stride. Finally, these three bottleneck blocks and a skip connection are concatenated to each other using an addition layer.

#### 2.3.5 Block 4

**Figure 5:**
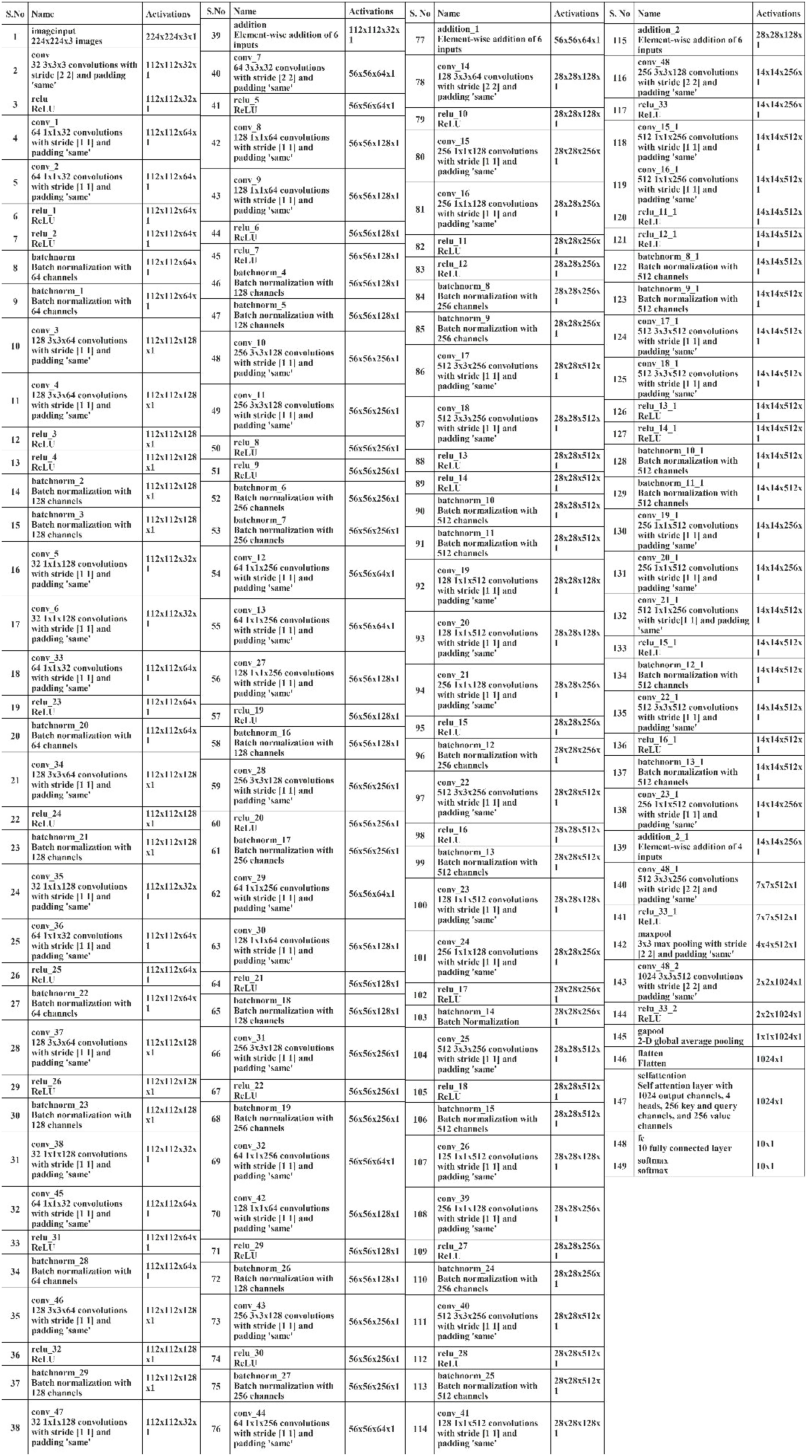
Tabular architecture of proposed Bottleneck Residual with Self-Attention (BRwSA).

A convolutional layer has been added after the fourth block. The depth size of this layer is 512, with a filter size of 3×3 and stride 2. A ReLu activation layer has been added to each convolutional layer. After that, a max-pooling layer of filter size 3×3 and stride two is included. Subsequently, a convolutional layer of depth size 1024 is added, and the filter and stride values are the same as the previous convolutional layer. A global average pool layer is added after the convolutional layer to control the number of parameters and weights, followed by a flattened layer. The output channel of the flattened layer is passed to the self-attention layer. This layer extracts more informative and in-depth information about the disease leaf region. Finally, a fully connected softmax and classification output layers have been added that complete this network. **Figure 5** illustrates the proposed layers’ weights and activation. There are 149 layers overall and 23.6 M training parameters in total.

### 2.4 Proposed inverted bottleneck residual with self-attention CNN

The proposed Inverted Bottleneck Residual with Self-Attention (IBRwSA) architecture is based on inverted bottleneck blocks, in which the channels are expanded first and then squeezed. The inverted bottleneck block with depthwise separable convolution is more efficient than the original. Moreover, the grouped convolutions allow us to build wider networks by replicating the modular blocks of filter groups. Hence, by using this structure, we can increase the network capacity without compromising computation efficiency. In the inverted bottleneck, we follow the filter size in the sequence: 1×1, 3×3 for 2-D grouped convolution layer, and 1×1. For channel-wise separable convolution, the grouped convolutional layer is employed. The final 1×1 layer increases the feature count, which makes it more enticing and richer.

#### 2.4.1 Block 1

**Figure 6:**
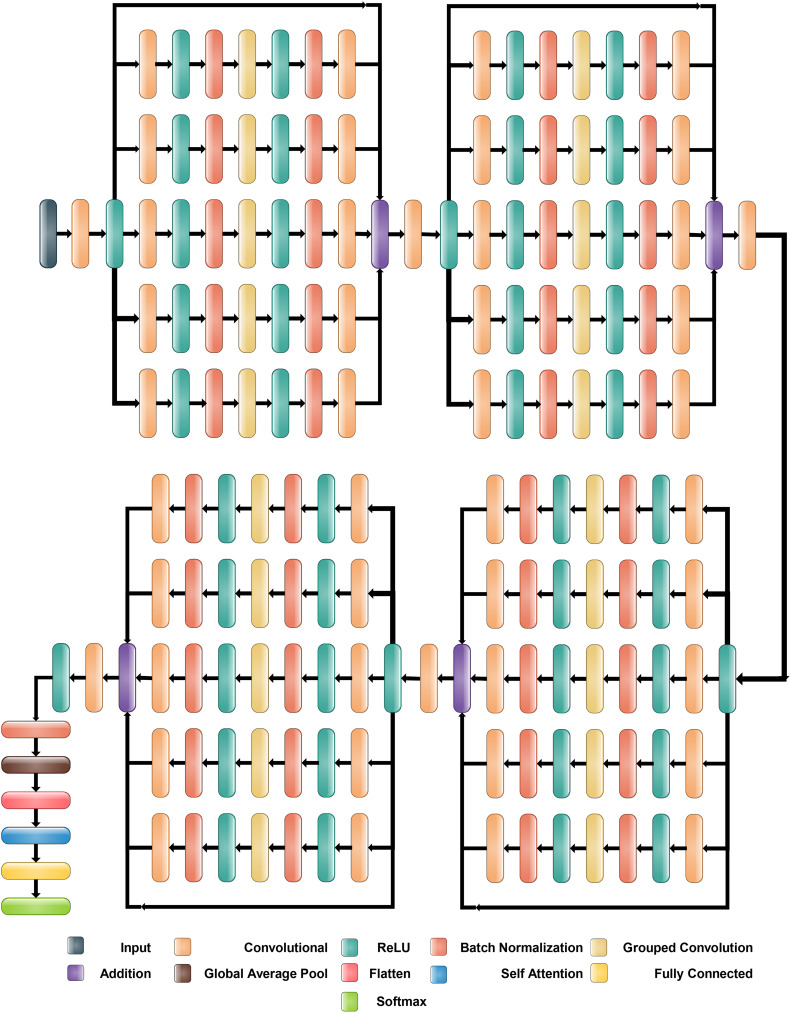
Proposed Inverted Bottleneck Residual with Self-Attention (IBRwSA) architecture.

**Figure 6** illustrates the proposed IBRwSA architecture. This figure includes four parallel residual blocks that follow the mechanism of the inverted bottleneck. In each block, a skip connection is also included that is concatenated at an additional layer with other paths. An input size of 227×227 with a depth size of 3 is considered in the first layer. After that, a convolutional layer has been added of depth size 32, filter size 3×3 and stride 2. The ReLu activation layer is included after each convolutional layer in the entire network.

The first parallel residual inverted bottleneck block consists of five paths and one skip connection. In each path, seven layers have been included, such as two convolutional, two ReLu, 2 batch normalizations, and one group convolutional. The first convolutional layer depth size is 64, whereas the filter size is 1×1, and stride 1. This layer follows the ReLU activation and batch normalization layers, respectively. After that, a 2-D grouped convolution layer was added using a channel-wise approach. The filter size of this layer is 3×3 and stride 1. The ReLu activation is added, followed by a batch normalization layer. Another convolutional layer has been inserted with a depth size of 32 and a filter size of 1×1. The remaining paths in this block are considered the same pattern, including depth size, filter size, and stride value. Finally, all paths of this block are added into an additional layer with a skip connection.

#### 2.4.2 Block 2

In this block, a convolutional layer is added before the start of the parallel paths. The depth size of the convolutional layer is 64, with a filter size of 3×3 and stride 2. A ReLu activation layer is included after the convolutional layer. After that, a new block is added that includes five parallel paths, and a sequence of layers is added in each path. The first convolutional layer of this block has a depth value of 128, a filter size of 1×1, and a stride one. After this, the ReLU activation and batch normalization layers are added, which is further followed by a grouped convolutional layer in channel-wise 3×3 and stride 1. The ReLU activation and batch normalization layers are added after the grouped convolutional layer. Finally, the last convolutional layer is added 64-depth, with a filter size 1×1 and a single stride. These five paths and one skip connection are connected in an additional layer. Subsequently, a convolutional layer is added with a depth of 128, stride value of 2×2, and filter size of 3, followed by a ReLU activation layer.

#### 2.4.3 Block 3

The third block follows the same pattern as the previous block, containing five inverted bottleneck blocks appended in parallel, each consisting of Convolution 1×1 with a depth of 256, 2-D Grouped convolution with channel 3×3, and Convolution 1×1 with a depth of 128. All five parallel paths are connected with a skip connection in the addition layer. Later, a convolution layer is added consisting of 256 depth size, 3×3 filter size, and stride 2. A ReLU activation layer is also included after this layer.

#### 2.4.4 Block 3

This block follows a similar pattern, like several layers, except for depth size. Five inverted bottleneck paths are included, where each path consists of a convolutional layer of depth value 512, filter size 1×1, and single stride. The grouped convolutional layer with channel-wise added filter size is 3×3, which finally ended with another convolutional layer of depth size 256. These five inverted bottleneck paths and a skip connection are concatenated using an addition layer.

**Figure 7:**
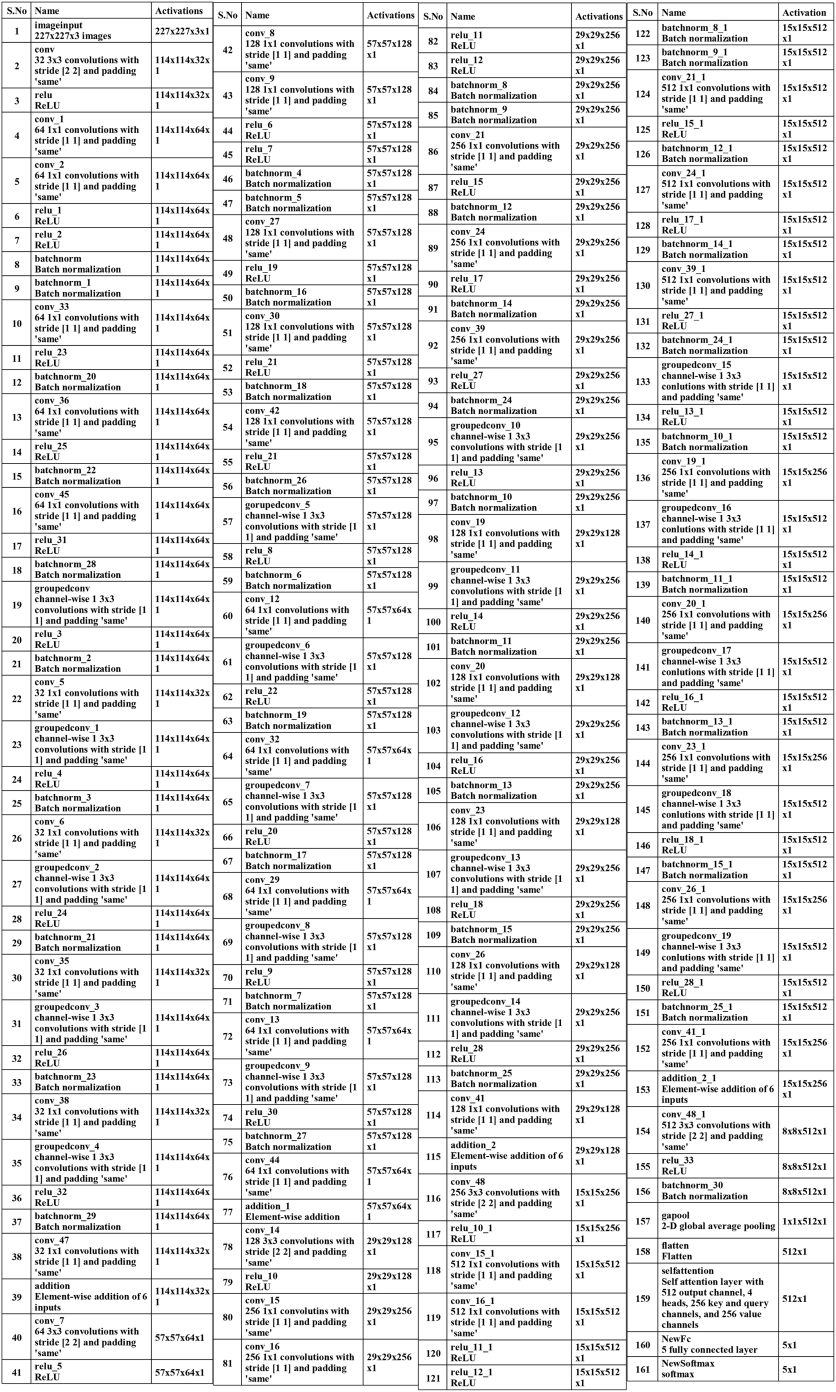
Detail description four-block inverted bottleneck layered model.

Subsequently, a 512-depth convolution layer with 3×3 filter size and stride two is incorporated, followed by a ReLU activation layer and batch normalization layer. The global average pool layer is added, followed by flattened, self-attention, fully connected, and Softmax layers. The proposed model is also presented in the tabular form in **Figure 7**. This network consists of 161 layers and the 3.9M total learnable.

### 2.5 Models training

The training process of the proposed model is presented in this subsection. In the training process, a 60:10:30 strategy was conducted, which means 60% of the images of the selected datasets were employed for the training, 10% of data were used for the validation during the learning process and the remaining images were utilized for testing the proposed models. Several hyperparameters have been employed in the training, such as learning rate, momentum, optimizer, mini-batch size, and regularization factor. These values are initialized using a human learning optimization algorithm (algorithm explained in the testing section). The best-selected value of the initial learning rate of 0.0002, the momentum of 0.702, the mini-batch size value of 64, stochastic gradient descent used as an optimizer, and 100 epochs. After that, the trained models are employed in the feature extraction and classification testing phase.

### 2.6 Proposed framework testing

In the testing phase of the proposed framework, the following steps are considered: i) employing a trained model and extracting deep features from the testing data; ii) testing features are passed to the fusion function for features concatenation; iii) features are selected using improved human learning algorithm, and iv) selected features are classified using machine learning algorithms. As shown in **Figure 2**, the testing process is based on the steps mentioned above. In the first step, features are extracted from the trained models (Cucumber and Apple datasets separately) and fused using a concatenation approach. The self-attention layer is employed for the feature extraction for both models and obtained a feature vector of N × 1024 and N × 512, respectively. After the concatenation, the size of the fused vector is N × 1536, where N denotes the number of testing samples in each dataset. Fused features are optimized using an improved human learning algorithm following the final classification process.

#### 2.6.1 Features fusion and optimization

Features extracted from the self-attention layer of both proposed models are fused using a concatenation function. Considering we have two feature vectors of dimensional N × 1024 and N × 512, the fused vector size will be N × 1536. However, a thorough analysis was conducted, and it was observed that a few features are not required for the classification process. Also, there are several redundant features; therefore, we implemented an improved human learning optimization algorithm for the best feature selection.

##### 2.6.1.1 Human Learning Optimization (HLO)

Human learning, by nature, is a repetitious optimization process (Wang L. et al., 2015). Activities such as playing baseball or learning to dance are improved and mastered by repeatedly learning, similar to the global optima iteration of meta-heuristics searching (Wang L. et al., 2015). In this work, the improved HLO is used based on the four learning operators: the individual learning (IL) operator, social learning (SL) operator, random exploration learning (REL) operator, and relearning (RL) operator. Mathematically, the algorithm is defined in the following steps.

##### 2.6.1.2 Initialization

HLO uses a binary coding system to solve problems, each bit resembling the fundamental piece of information. Thus, in Equation 3, a candidate solution is initialized with “0” or “1,” also known as binary strings, while randomly assuming that there was no prior knowledge of the issue.


(3) $$\begin{array}{l} X_{I}=\left[X_{I1}\;\;X_{I2}\;\ldots \;\;X_{IJ}\;\ldots \;\;X_{Im}\right],\\ \;\;1\leq I\leq n,\;1\leq J\leq m \end{array}$$

Where $I^{th}$ individual is $X_{I}$ , the number of individuals in the population is denoted by n , and the number of components contained in the knowledge m can also be known as the dimensions of solutions used to initialize each person. (Equations 4, 5) presents the HLO population upon initialization.


(4) $$x=\left[\begin{array}{c} X_{1}\\ \begin{array}{c} X_{2}\\ \vdots \\ X_{I} \end{array}\\ \begin{array}{c} \vdots \\ X_{n} \end{array} \end{array}\right]=\left[\begin{array}{c} \begin{array}{ccc} X_{11} & X_{12} & \cdots \\ X_{21} & X_{22} & \cdots \\ \vdots & \vdots & \end{array}\;\;\;\begin{array}{ccc} X_{1J} & \cdots & X_{1m}\\ X_{2J} & \cdots & X_{2m}\\ \vdots & & \vdots \end{array}\\ \begin{array}{ccc} X_{I1} & X_{I2} & \cdots \\ \vdots & \vdots & \\ X_{n1} & X_{n2} & \cdots \end{array}\;\;\begin{array}{ccc} X_{IJ} & \cdots & X_{Im}\\ \vdots & & \vdots \\ X_{nJ} & \cdots & X_{nm} \end{array} \end{array}\right]$$

(5) $$X_{IJ}\in \left\{0,1\right\},\;\;1\leq I\leq n,\;\;1\leq J\leq m$$

##### 2.6.1.3 Learning Operators

There are four learning operators for HLO. Each one is described below:

##### 2.6.1.4 Individual learning (IL) operator

The ability to construct knowledge about external influences and sources by personal reflection can be defined as individual learning. Through HLO, an individual learns how to solve problems by using their own experiences, which are stored in the Individual Knowledge Database (IKD), represented by (Equations 6–8).


(6) $$X_{IJ}=IK_{IPJ}$$

(7) $$IKD_{I}=\left[\begin{array}{c} ikd_{I1}\\ \begin{array}{c} ikd_{I2}\\ \vdots \\ ikd_{Ip} \end{array}\\ \begin{array}{c} \vdots \\ ikd_{Ig} \end{array} \end{array}\right]=\left[\begin{array}{l} ik_{I1\;1}\;ik_{I1\;2}\;\cdots \;ik_{I1\;J}\;\cdots \;ik_{I1\;m}\\ ik_{I2\;1}\;ik_{I2\;2}\;\cdots \;ik_{I2\;J}\;\cdots \;ik_{I2\;m}\\ \;\vdots \;\vdots \;\vdots \;\vdots \;\\ ik_{IP\;1}\;ik_{IP\;2}\;\cdots \;ik_{IP\;J}\;\cdots \;ik_{IP\;m}\\ \;\vdots \;\vdots \;\vdots \;\vdots \;\\ ik_{Ig\;1}\;ik_{Ig\;2}\;\cdots \;ik_{Ig\;j}\;\cdots \;ik_{Ig\;m} \end{array}\right]$$

(8) 1≤I≤n,  1≤P≤g,  1≤J≤m

Where the IDK of person I is represented by $\;IKD_{I}$ , the best answer for each person I is represented by $ikd_{IP}$ , and a random number P decides which individual in the IKD is chosen for IL. The size of the $\;IKD_{S}$ is represented by g .

##### 2.6.1.5 Social learning (SL) operator

The transfer of skills and knowledge among individuals through direct or indirect interaction is called social learning. In order to have an effective search function, HLO mimics the SL mechanism. Each HLO researcher examines the social knowledge included in the Social Knowledge Database (SKD) with a certain degree of probability. Similar to how human learning produces new solutions as presented in (Equations 9–11).


(9) $$X_{IJ}=SK_{QJ}$$

(10) $$SKD=\left[\begin{array}{c} skd_{1}\\ \begin{array}{c} skd_{2}\\ \vdots \\ skd_{p} \end{array}\\ \begin{array}{c} \vdots \\ skd_{h} \end{array} \end{array}\right]=\left[\begin{array}{l} sk_{1\;1}\;sk_{1\;2}\;\cdots \;sk_{1\;J}\;\cdots \;sk_{1\;m}\\ sk_{2\;1}\;sk_{2\;2}\;\cdots \;sk_{2\;J}\;\cdots \;sk_{2\;m}\\ \;\vdots \;\vdots \;\vdots \;\vdots \;\\ sk_{Q\;1}\;sk_{Q\;2}\;\cdots \;sk_{Q\;J}\;\cdots \;sk_{Q\;m}\\ \;\vdots \;\vdots \;\vdots \;\vdots \;\\ sk_{h\;1}\;sk_{h\;2}\;\cdots \;sk_{h\;J}\;\cdots \;sk_{h\;m} \end{array}\right]$$

(11) 1≤Q≤h,  1≤J≤m

Where the size of SKD is h and the $Q^{th}$ social knowledge in SKD is represented by $SKD_{Q}$ , that is, the newly created candidate $X_{I}$ duplicates the relevant bit after selecting at random one of the better solutions kept in the SKD.

##### 2.6.1.6 Random exploration learning (REL) operator

The exploratory processes are characterized by unpredictability since the novel challenge is typically unknown beforehand. By using (Equation 12) with a specific probability to conduct out REL, HLO simulates these occurrences.


(12) $$X_{IJ}=RE\left(0,1\right)=\{\begin{array}{c} 0,\;\;rand<0.5\\ 1,\;\;else \end{array}$$

Where the random number rand value is between 0 and 1.

##### 2.6.1.7 Relearning operator

This could potentially assist HLO in breaking free from local optima and achieving improved performance, similar to individuals relearning using a novel strategy to get past the bottleneck.

##### 2.6.1.8 Updating the IKD and SKD

After people complete learning in each generation, the fitness of a new alternating solution is determined using a pre-established fitness function, which is presented in (Equation 13).


(13) COST=ρ∗ERROR+ς∗(number of selected features/maxof features)

Where  ρ is initialized as 0.82,  ς is initialized as 0.02, and the error is defined in (Equation 14).


(14) ERROR=1−ACCURACY

(Equation 15) states the specific rates at which REL, SL, and IL are performed to produce new solutions.


(15) $$X_{IJ}=\{\begin{array}{c} \begin{array}{cc} RE\left(0,1\right), & 0\leq rand<PR \end{array}\\ \begin{array}{cc} IK_{IPJ}, & \;\;PR\leq rand<PI \end{array}\\ \begin{array}{cc} SK_{QJ},\;\;\;\;\;\;\;\;\;\;\; & \;\;\;\;else \end{array} \end{array}$$

The rate of SL and IL is represented by (1−PI) and (PI−PR) respectively, here PR stands for the likelihood of REL. The information included in the IKD is removed using the relearning operator if a person’s learning gets caught in a bottleneck. This allows the individual to resume learning without being influenced by prior experiences. Until the termination requirements are satisfied, the HLO update operation and learning operators are repeatedly performed. The **Algorithm 1** is updated with the Bayesian inference learning for the final selection (Zhang et al., 2023).

Algorithm 1 **Proposed feature selection algorithm.**

**Table:** 

**Input**:	Feature Vector ← $FV_{i}$
**Output:**	Optimal vector $\leftarrow {\tilde{F}}_{K}$
Where (i=1 ;N)	
**Step:1**	**Initialize Parameters** Number of Solution ← 10 Interaction ← 100 Number of ‘ k ‘ in K-Nearest Neighbor ← 10 Ratio of validation data ( ho ) ← 0.3 pi← 0.85 pr← 0.1
**Step:2**	Compute Fitness and generate initial IKD and SKD using Eq. (6) to (11).
**Step:3**	If (N completed): { $\leftarrow {\tilde{F}}_{K}$ } Else: Generate new solution Computer fitness using eq. (12) Update IKD and SKD using eq. (13) and (14)
**Step:4**	If (rewrite required): {Clear IKD using eq. (15) and reach step 3} Else: Step 3 for terminations

### 2.7 Shallow neural network classifier

**Figure 8:**
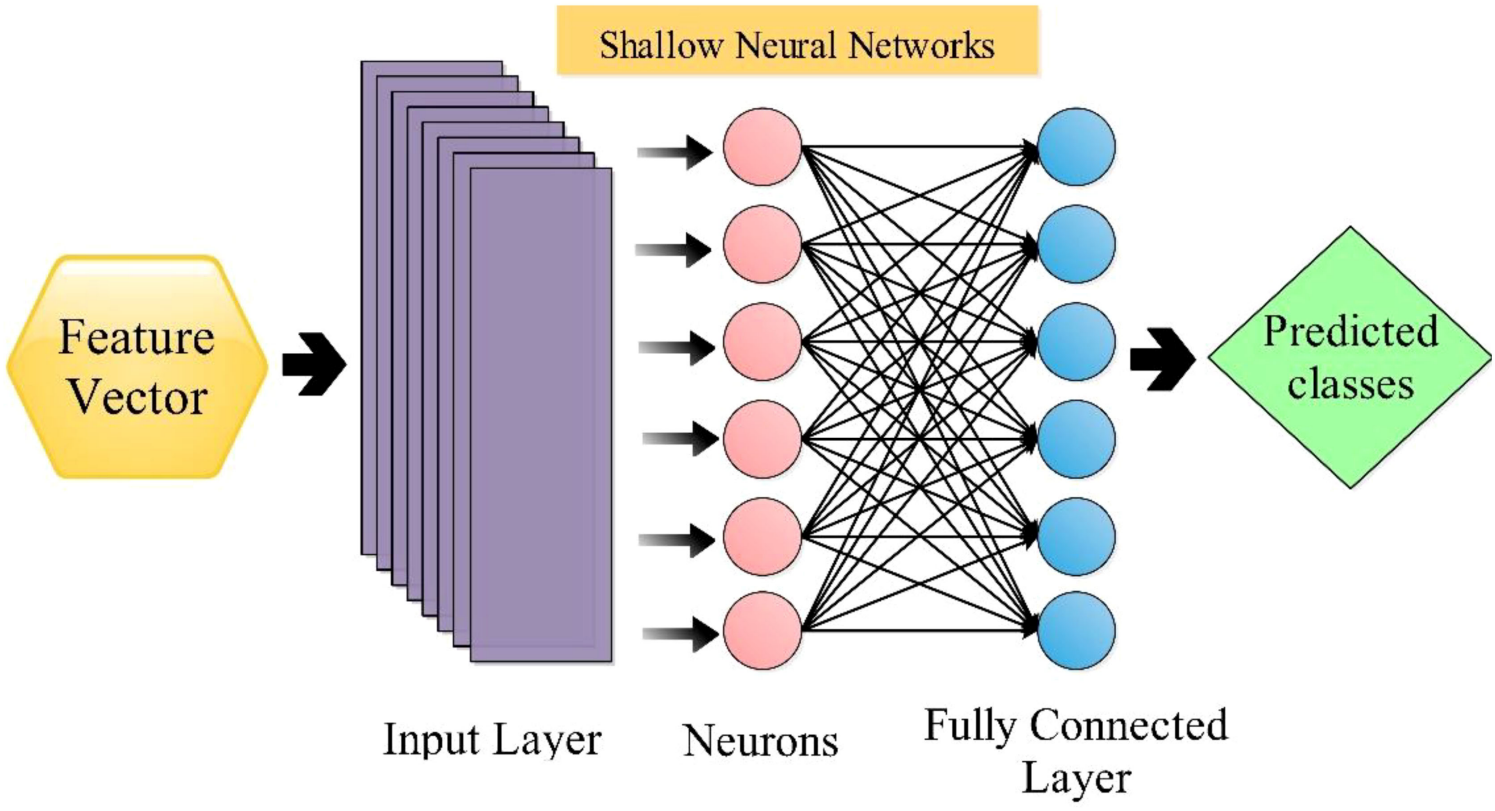
Shallow wide neural network classifier for the classification.

The shallow wide neural network [SWN (Gavhale and Gawande, 2014)] classifier is utilized in this work to classify selected features. The SWNN classifier consists of one fully connected layer with ten neurons in the input layer. The architecture of SWNN is shown in **Figure 8**. Followed by the next classifier, the medium neural network (MN^2^) is composed of one fully connected layer, with the layer size being 25. Next comes the Narrow Neural Network (N^3^), Bilayered Neural Network (BiN^2^), and Trilayered Neural Network (TiN^2^); that input layer size was 100 and included two hidden layers (fully connected). These classifiers are employed for the classification comparison with SWN^2^.

## 3 Results and discussion

The results of this work’s experiment are explained using tables and confusion matrices in the following section.

### 3.1 Experimental environment

The datasets used for this experiment are the apple and cumber, as discussed in section 2.1. 70% of the images were used for the training procedure, and the remaining 30% were employed for the testing. The classifiers are applied after extracting features of both models, after the fusion of the feature, and after the optimization is applied to both datasets. A 10-fold cross-validation approach has been utilized in the entire experimental process. Several neural network classifiers and performance metrics were used throughout the validation phase, including accuracy, processing time, f1 score, tpr, PPV, FPR, and area under the curve. The framework was simulated on MATLAB 2023b using a Personal Computer with 128GB RAM and 12GB Graphics Card RTX 3060.

### 3.2 Apple dataset results

**Figure 9:**
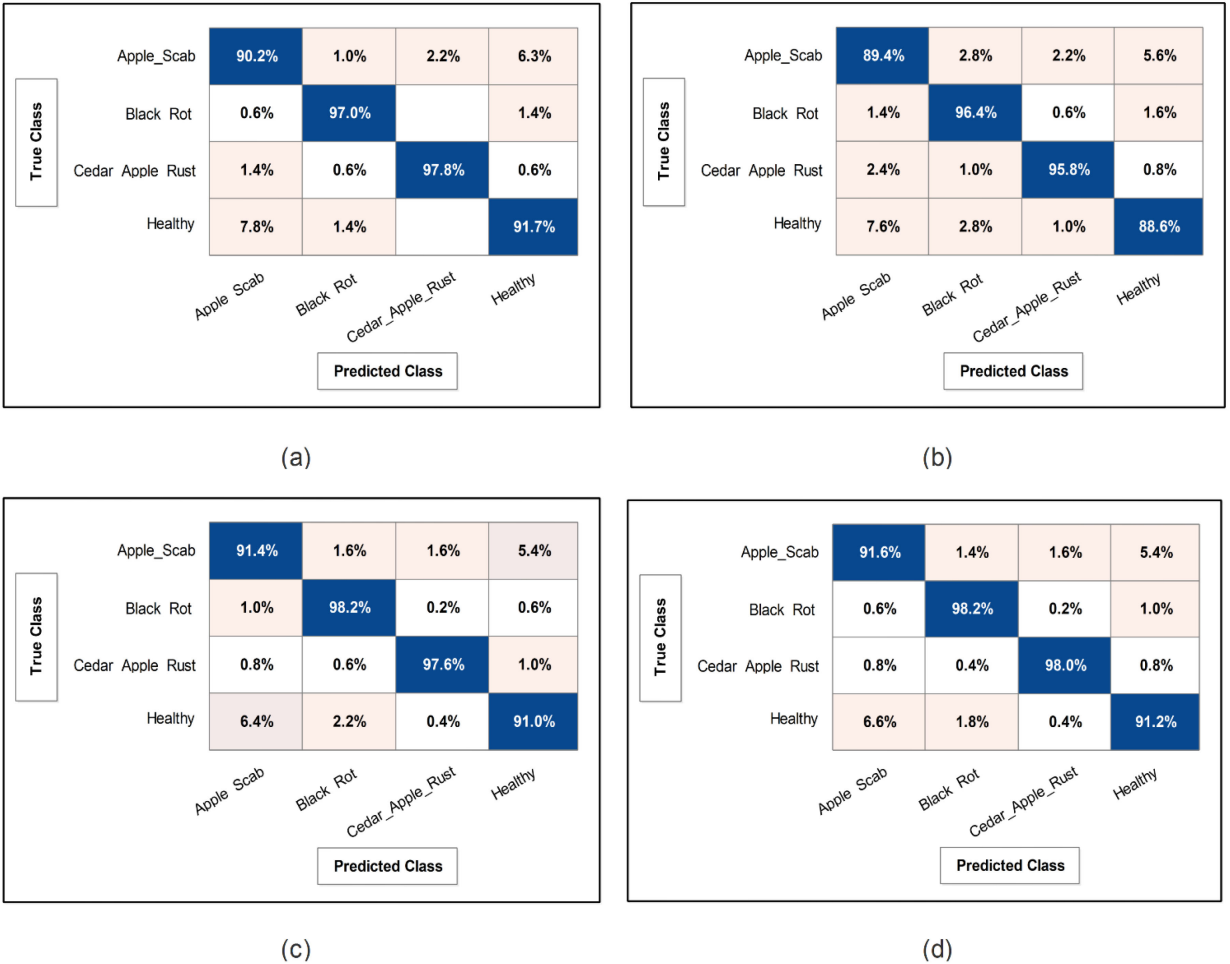
Confusion matrix of SWNN classifier for each performed experiment using Apple dataset. **(A)** Confusion matrix of proposed BRwSA architecture, **(B)** confusion matrix of proposed IBRwSA architecture, **(C)** confusion matrix of fused features, and **(D)** confusion matrix of proposed selected features.

This subsection presents the Apple dataset results as numerical and confusion matrices. **Table 2** presents the proposed classification results. The first part (a) presents the results of proposed Bottleneck Residual with Self-Attention (BRwSA) architecture in this table. The SWN^2^ classifier obtained the best accuracy of 94.2%, whereas the execution time was 24.745 (sec). The TPR value of this classifier is 94.2, the PPV value is 94.175, the F1-Score value is 94.187, and AUC is 0.99175, respectively. The other shallow classifiers, such as SN3 and SMN2, achieved 93.8 and 93.8% accuracy, respectively. A small decline of 0.4% is noted in the performance of these classifiers compared to SWN^2^. The confusion matrix of this experiment is illustrated in **Figure 9A**, which can be employed to verify SWN^2^ results. In this figure, the correct prediction rate of each class is 90.2, 97.0, 97.8, and 91.7%, respectively. The testing time of the classification process is also noted, and the lowest recorded computational time is 21.118 sec for the SMN^2^ classifier.

**Table 2:** Classification results of the proposed framework on Apple Dataset.

*(a)* Classification results of proposed Bottleneck Residual with Self-Attention (BRwSA) architecture							
Classifiers	TPR (%)	PPV (%)	F1 Score (%)	FPR	AUC	ACC (%)	Time (Sec)
N^3^	93.75	93.75	93.75	0.0208	0.9767	93.8	34.048
MN^2^	93.85	93.85	93.85	0.0204	0.9888	93.8	21.118
**SWN^2^**	**94.2**	**94.17**	**94.18**	**0.0193**	**0.99175**	**94.2**	**24.745**
BiN^2^	93.75	93.75	93.75	0.0208	0.9675	93.8	37.666
TiN^2^	93.7	93.675	93.687	0.02097	0.9755	93.7	44.681
*(b)* Classification results of proposed Inverted Bottleneck Residual with Self-Attention (IBRwSA) architecture							
N^3^	91.90	91.85	91.8749	0.02699	0.9615	91.9	48.148
MN^2^	92.05	92.025	92.0374	0.0265	0.983	92.0	40.226
**SWN^2^**	**92.55**	**92.55**	**92.55**	**0.0248**	**0.98515**	**92.5**	**55.245**
BiN^2^	91.6	91.6	91.6	0.0279	0.9678	91.6	54.529
TiN^2^	91.35	91.35	91.35	0.02883	0.9607	91.3	66.428
*(c)* Classification results of fused features							
N^3^	94	94	94	0.01995	0.9809	94	56.359
MN^2^	94.1	94.1	94.1	0.01966	0.9909	94.1	51.867
**SWN^2^**	**94.55**	**94.55**	**94.55**	**0.01813**	**0.9926**	**94.5**	**57.711**
BiN^2^	93.8	93.8	93.8	0.02065	0.9798	93.8	44.631
TiN^2^	94.25	94.25	94.25	0.01914	0.97735	94.2	53.681
*(d)* Classification results of proposed optimization algorithm							
N^3^	94.175	94.25	94.212	0.0194825	0.9754	94.2	21.401
MN^2^	94.55	94.55	94.55	0.018125	0.99177	94.5	17.503
**SWN^2^**	**94.75**	**94.75**	**94.755**	**0.01748**	**0.9928**	**94.8**	16.859
BiN^2^	93.9	94.05	93.974	0.0198	0.9803	94.0	**13.208**
TiN^2^	93.85	93.85	93.85	0.02048	0.97765	93.8	17.917

Bold denotes the best accuracy values.

The second part of this table presents the classification results of proposed Inverted Bottleneck Residual with Self-Attention (IBRwSA) architecture. The SWN^2^ classifier obtained the highest accuracy of 92.5%, with an execution time of 55.245 sec. The TPR value of this classifier is 92.55, a PPV value of 92.55, an F1-Score value of 92.55, and an AUC of 0.98515, respectively. The rest of the classifiers obtained an accuracy of 92.0, 91.9, 91.3, and 91.6%, respectively. The confusion matrix of the SWN^2^ classifier is illustrated in **Figure 9B**, which can be utilized to verify the computed TPR value. The correct prediction rates for each class in this figure are 89.4, 96.4, 95.8, and 88.6%, respectively. Also, the computational time of each classifier is noted, and SMN^2^ has the lowest reported time of 40.226 sec.

Compared to the results of both proposed architectures, it is noted that the BRwSA model shows an improvement in accuracy of 1.7%. Moreover, the TPR and PPV of this model are better than those of IBRwSA. In addition, the proposed BRwSA architecture executed faster than the IBRwSA. Features fusion of both models, the accuracy and TPR rates are improved. In the third part of this table, the fusion results are presented. After the fusion, the maximum obtained accuracy was 94.5%, whereas the execution time was 57.711 sec. This classifier’s TPR, PPV, F1-Score, and AUC values are 94.55, 94.55, 94.55, and 0.9926, respectively. A confusion matrix is also illustrated in **Figure 9C**, representing that each class’s correct prediction rate is 91.4, 98.2, 97.6, and 91.0%. The computational time of the fusion process is increased; however, the minimum noted time is 44.631 sec using the BN^2^ classifier. Compared to the fusion results with individual deep learning models, it is observed that the accuracy is improved; however, the time is also increased.

The proposed optimization algorithm has been performed to improve the accuracy further and reduce the computational time in the testing process. The results are noted in the last part of **Table 2**. The SWN^2^ shows an improved accuracy of 94.8%, whereas the execution time is 13.208 sec. There are a few other performance measures, such as TPR value of 94.75, PPV value of 94.75, F1-Score of 94.755, and 0.99.28 AUC value. The accuracy of the other shallow classifiers is 94.2, 94.5, 94.0, and 93.8%, respectively. Based on these values, accuracy improves after the optimization process. **Figure 9D** shows the confusion matrix for the shallow wide classifier, which may be used to confirm the TPR value. The image displays the accurate prediction rate of each class as follows: 91.6, 98.2, 98.0, and 91.2%. Compared with previous experiments, the optimization process shows better performance. In addition, the minimum computational time is 13.208 (sec), which is significantly reduced.

### 3.3 Cucumber dataset results

**Figure 10:**
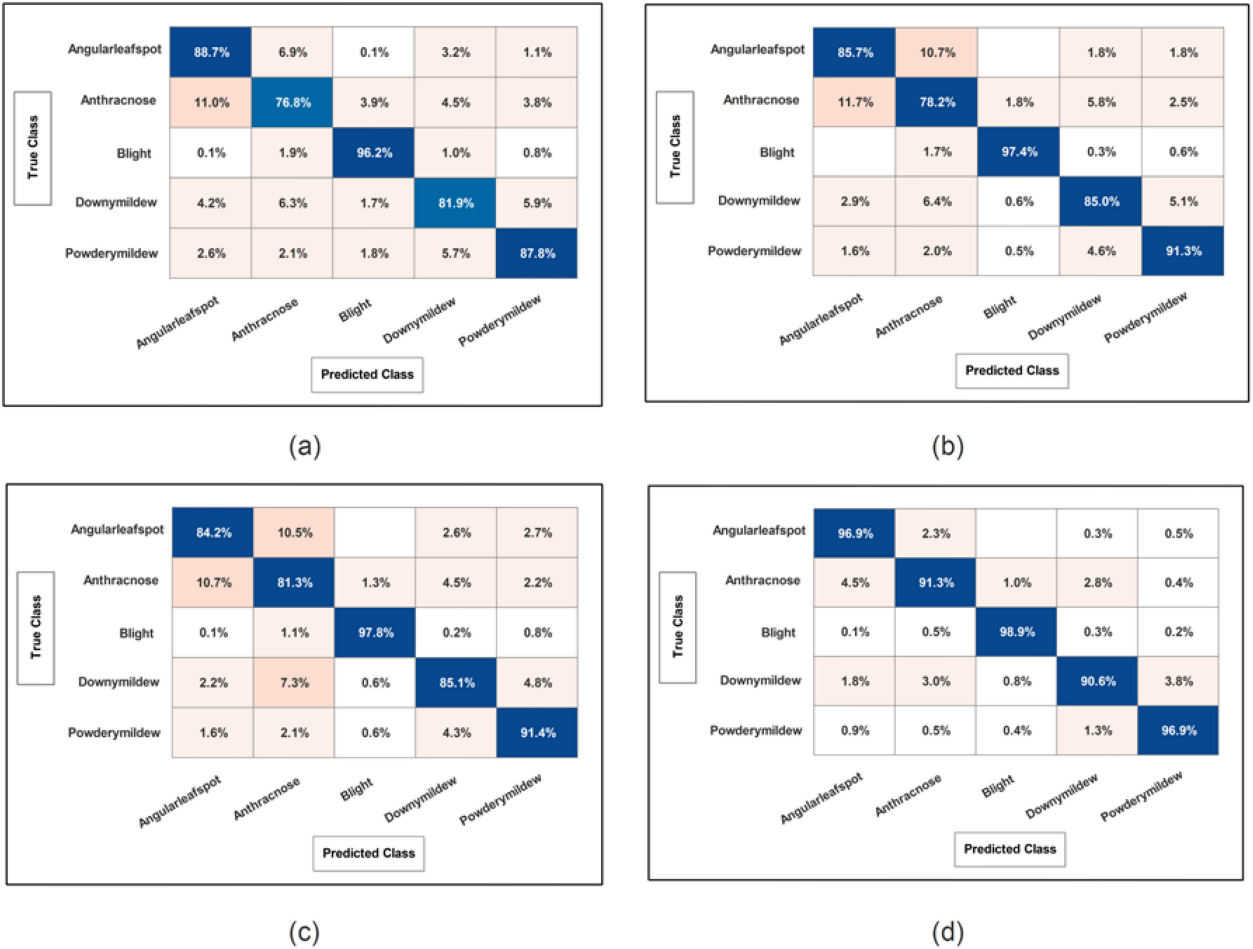
Confusion matrix of SWNN classifier for each performed experiment using Cucumber dataset. **(A)** Confusion matrix of proposed BRwSA architecture; **(B)** confusion matrix of proposed IBRwSA architecture; **(C)** confusion matrix of fused features, and **(D)** confusion matrix of proposed selected features.

Cucumber dataset results are presented in this subsection. **Table 3** presents the detailed numerical results of the proposed framework using the cucumber dataset. In the first part of this table, the proposed BRwSA architecture results show the maximum obtained accuracy of 86.3% for the SWN^2^ classifier, whereas the execution time is 47.699 sec. The TPR value of this classifier is 86.28, the PPV value is 86.28, the F1-Score value is 86.392, and AUC is 0.95402, respectively. The other shallow classifiers obtained 82.6, 85.7, 83.7, and 81.47% accuracy, respectively. **Figure 10A** illustrates the confusion matrix of SWN^2^ for this experiment that can be utilized to verify the TPR value of SWN^2^. When computational time is noted, the SMN^2^ required a minimum time of 15.501 sec.

**Table 3:** Proposed framework classification results using the Cucumber dataset.

Classification results of proposed Bottleneck Residual with Self-Attention (BRwSA) architecture							
Classifiers	TPR	PPV	F1 Score	FPR	AUC	ACC	Time
N^3^	82.64	82.58	82.6099	0.0434	0.90642	82.6	96.49
MN^2^	85.62	85.66	85.639	0.03585	0.94302	85.7	15.501
**SWN^2^**	**86.28**	**86.2**	**86.239**	**0.0343**	**0.95402**	**86.3**	**47.699**
BiN^2^	83.72	83.6	83.6599	0.0407	0.91952	83.7	80.36
TiN^2^	81.36	81.26	81.309	0.0566	0.91064	81.47	61.3
*(b)* Classification results of proposed Inverted Bottleneck Residual with Self-Attention (IBRwSA) architecture							
N^3^	84.02	84	84.01	0.0399	0.92196	84.0	241.93
MN^2^	85.58	85.6	85.899	0.03605	0.9617	85.6	60.799
**SWN^2^**	**87.52**	**87.5**	**87.51**	**0.0312**	**0.97054**	**87.5**	**105.96**
BiN^2^	84.24	84.26	84.25	0.0394	0.9299	84.2	196.58
TiN^2^	83.38	83.32	83.349	0.04155	0.9249	83.4	203.98
*(c)* Classification results of fused features							
N^3^	84.08	83.98	84.02	0.0398	0.92166	84.1	157.99
MN^2^	86.4	86.4	86.4	0.034002	0.9657	86.4	72.057
**SWN^2^**	**87.96**	**87.98**	**87.97**	**0.0301**	**0.9726**	**88.0**	**125.2**
BiN^2^	83.34	83.26	83.299	0.04165	0.91624	83.3	248.66
TiN^2^	83.76	83.7	83.73	0.0406	0.92694	83.8	293.38
*(d)* Classification results of proposed feature selection algorithm for Cucumber dataset							
N^3^	90.74	90.7	90.72	0.02315	0.96058	90.7	53.91
MN^2^	93.02	93.02	93.02	0.01745	0.97874	93.0	31.962
**SWN^2^**	**94.92**	**94.92**	**94.92**	**0.0127**	**0.98672**	**94.9**	**37.925**
BiN^2^	90.94	90.9	90.92	0.02265	0.9587	90.9	74.79
TiN^2^	90.84	90.78	90.80	0.0229	0.9621	90.8	105.84

Bold denotes the best accuracy values.

In the second part of this table, IBRwSA architecture results are presented. The SWN^2^ classifier obtained the highest accuracy of 87.5%, with an execution time of 105.96 sec. The TPR value of this classifier is 87.52, a PPV value of 87.5, an F1-Score value of 87.51, and an AUC value of 0.87054, respectively. The rest of the classifiers mentioned in this table obtained 85.6, 84.0, 83.4, and 84.2% accuracy, respectively. The confusion matrix is also presented in **Figure 10**, which can be used to verify the TPR rate of this classifier. Compared to the performance of this architecture with the proposed BRwSA, there is a slight reduction in accuracy, and an increase in time is noted.

To improve the performance of this dataset, we performed feature fusion. Results are discussed in **Table 3**(c), which shows the improvement in accuracy. The obtained accuracy after the fusion process is 88.0%, whereas the time is increased to 125.2 sec. To reduce the time and maintain the classification accuracy, we performed an optimization algorithm and obtained the maximum accuracy of 94.9% on the SWN^2^ classifier. The computational time of this classifier is 37.925 sec, which is significantly reduced. The TPR value of this classifier is 94.92, the PPV value is 94.92, the F1-Score is 94.92, and 0.98672 is the AUC value. The accuracy of the other shallow classifier is 90.7, 93.0, 90.9, and 90.8%, respectively. **Figure 10D** illustrates the confusion matrix of the SWN^2^ classifier that can be utilized to verify the TPR value. The correct prediction value of each class after the optimization process reaches 96.9, 91.3, 98.9, 90.6, and 96.9%, respectively. Overall, the optimization step improved the accuracy and reduced the computational time.

### 3.4 Ablation studies

**Figure 11:**
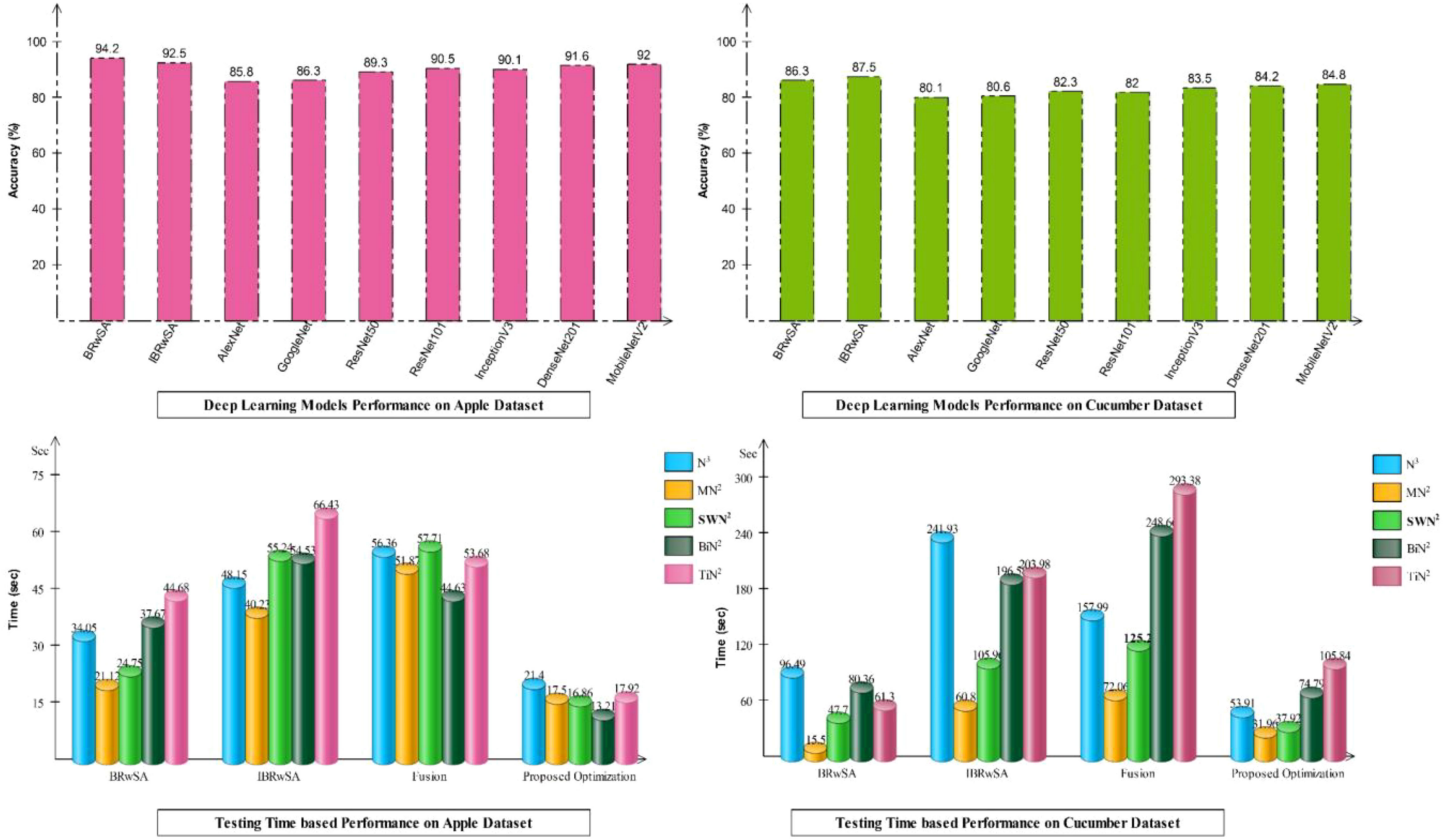
Analysis of proposed models and entire framework based on accuracy and testing time.

Detailed ablation studies of the proposed framework are described here based on the following points: performance of pre-trained models and proposed networks, time comparison, and comparison with recent SOTA techniques. The proposed framework consists of four important steps: BRwSA architecture, IBRwSA architecture, a fusion of features of both architectures and the selection of best features using an improved HLO algorithm. Results are discussed in **Tables 2**, **3**, showing the accuracy improvement after the fusion and optimization process. Confusion matrices are also illustrated in **Figures 9**, **10** are utilized to verify the TPR value of SWN2 for each experiment conducted for validation. From the results, we concluded that the proposed BRwSA architecture yielded better results than the IBRwSA architecture. In addition, this architecture contains fewer learning parameters and performs better than the pre-trained deep learning architecture (a comparison is conducted in **Figure 11**). In this figure, the upper part shows the accuracy of pre-trained and proposed architectures on the selected datasets separately. For the Apple dataset, the AlexNet model obtained an accuracy of 85.8%, and GoogleNet achieved 86.3%. The recent models, such as InceptionV3, DenseNet201, and MobileNetV2, obtained improved accuracies of 90.1, 91.6, and 92%, respectively. The proposed architectures obtained 94.2 and 92.5% accuracy, which is improved than the compared models. Similarly for the Cucumber dataset, the proposed architectures obtained 86.3 and 87.5% accuracy, whereas the pre-trained models obtained accuracy of 80.1, 80.6, 82.3, 82, 83.5, 84.2, and 84.8%, respectively. In the second part of this figure, testing time is plotted for each classifier. Four experiments are performed, and it is noted that the time is increased after the fusion process; however, this time is optimized using an improved HLO algorithm. Hence, time is significantly reduced after the optimization algorithm, which is this work’s strength.

**Figure 12:**
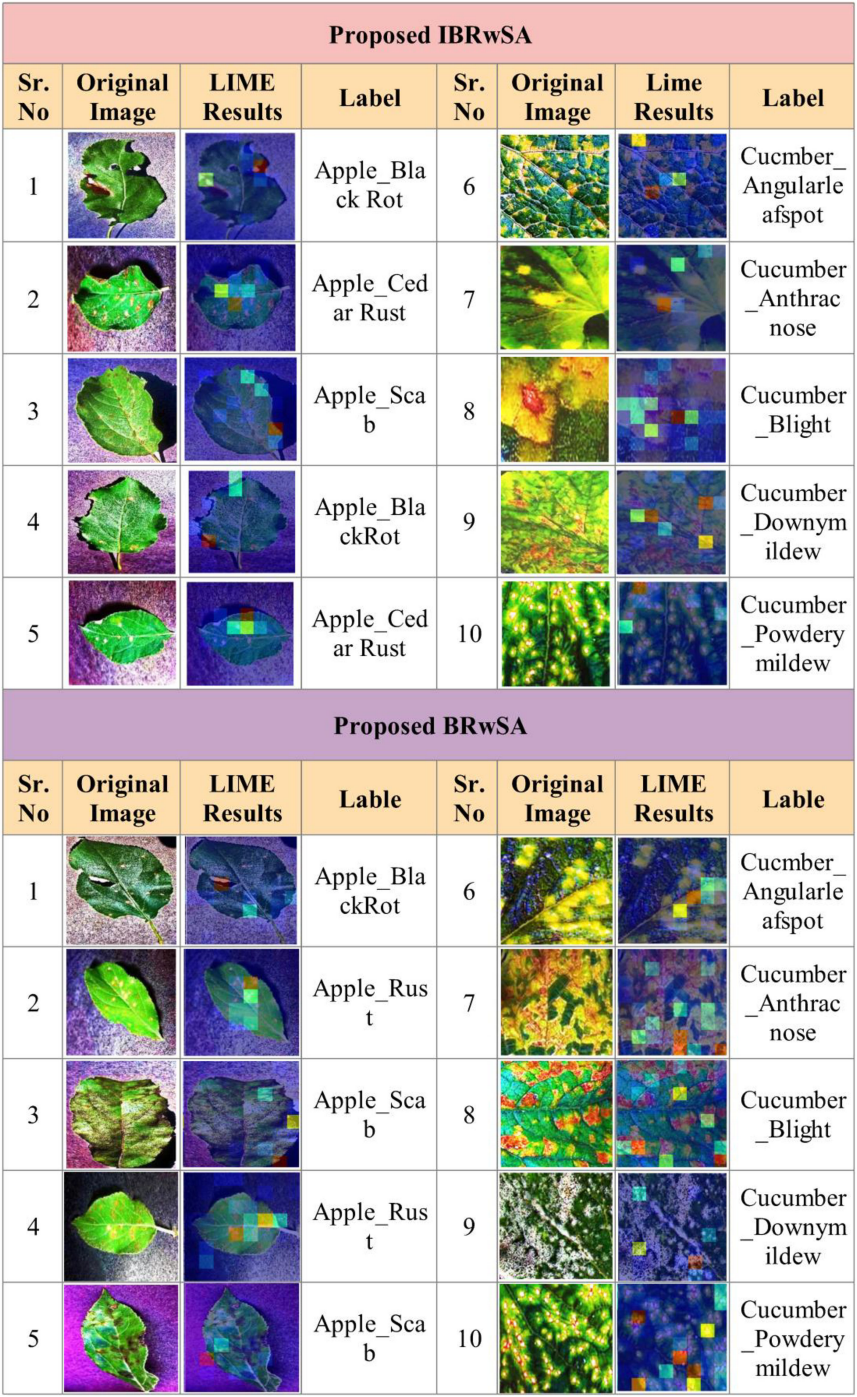
LIME based visualization results of the proposed IBRwSA and BRwSA architecture.

As shown in **Figure 11**, the proposed BRwSA architecture achieved better accuracy on the selected datasets than the proposed IBRwSA and pre-trained models; therefore, we employed the LIME technique as an explainable AI for the interpretation. The inside information of proposed IBRwSA BRwSA architecture is highlighted through LIME, as shown in **Figure 12**. The disease spots are highlighted with different colours based on the LIME interpretation. Except for all, the blue colour presents the healthy part in the image, which is wrongly identified as a diseased part.

**Table 4** summarises the proposed framework accuracy comparison with state-of-the-art (SOTA) techniques. In this table, a comparison is conducted based on datasets such as Apple and Cucumber. Authors (Zhong and Zhao, 2020) used an apple leaf image dataset and obtained a maximum accuracy of 93.71%. In (Bi et al., 2022), the ResNet152 model achieved the highest accuracy of 77.65%. Authors in (Khan et al., 2022) (Yu and Son, 2019), and (Kodors et al., 2021) used the Apple Leaf Image dataset and obtained an accuracy of 88.0, 84.3, and 87.0%, respectively. The proposed framework obtained an accuracy of 94.8%, which is better than the SOTA methods. Similarly, a comparison is conducted for the Cucumber dataset, and it is noted that the previous best reported accuracy was 94.7% by (Li et al., 2020) on cucumber leaf image dataset. The proposed framework obtained better accuracy of 94.9% using the Cucumber Private Dataset.

**Table 4:** Proposed framework comparison with recent state-of-the-art techniques on selected datasets.

Serial No.	Paper	Dataset	Accuracy
**1**	(Zhong and Zhao, 2020)	Apple Leaf Images Dataset	93.51, 93.31, and 93.71%
**2**	(Bi et al., 2022)	Apple Leaf Images Dataset based on shape, color, and disease count.	Mobile Net: 73.50 InceptionV3:75.59 ResNet 152: 77.65%
**3**	(Khan et al., 2022)	Apple Leaf Images Dataset	88%
**4**	(Yu and Son, 2019)	Apple Leaf Images Dataset	84.3%.
**5**	(Kodors et al., 2021)	Apple Leaf Images Dataset	87%
**6**	**Proposed Methodology**	Source: Plant village Dataset description: Apple Dataset: Apple Scab, Apple Cedar Rust, Black Rot and healthy	**94.8%**
(a) Cucumber Dataset			
**1**	(Kianat et al., 2021)	Cucumber Private Dataset	93.50%
**2**	(Li et al., 2020)	Cucumber Private Dataset	94.7%
**3**	(Mia et al., 2021)	Cucumber Private Dataset	Random Forest: 89.93%, MobileNetV2: 93.23%.
**4**	(Uoc et al., 2022)	Cucumber Private Dataset	80%
**5**	**Proposed Methodology**	Source: Privately Collected Dataset Dataset description: Cucumber Dataset: powdery mildew, anthracnose, blight, downy mild, and angular leaf spot	**94.9%**

Bold denotes the best accuracy values.

## 4 Conclusion

This work proposes a novel deep-learning framework with an improved HLO algorithm for apple and cucumber leaf disease classification. A contrast enhancement technique is proposed that increases the contrast of infected spots to help better feature extraction. Two novel deep learning architectures, BRwSA and IBRwSA, are proposed. Both architectures are trained on the selected datasets employed in the testing phase. Features are extracted from the self-attention layer and fused using a concatenation approach. Further, the fused features are optimized using an improved HLO algorithm. The selected features are finally classified using a shallow neural network classifier. The experimental process was conducted on two datasets and obtained improved 94.8 and 94.9% accuracy, respectively. Based on the detailed experiments, the following points are concluded:

▪ The contrast enhancement technique improved the contrast of the disease spot region, further helping to learn useful features.

▪ The inverted bottleneck model reduced a few important features in the convolutional layers compared to BRwSA; hence, the accuracy declined little and increased the number of learning parameters.

▪ Features are extracted from the self-attention layer and fused using a concatenation layer. The fusion process improved the accuracy, but computational time also jumped, which is the dark side of this step.

▪ Selection of best features using an improved HLO algorithm improved the accuracy and precision rate; however, another strong point was decreased computational time.

The limitation of proposed framework is the fusion process which increase the overall computation of the method. In future, we will propose activation based fusion and also will employ a light weight vision transformer for better learning. In addition to this, combine the selected datasets into a single dataset and then utilized for the training and testing. Based on this strategy, it can be easy to analyze the robustness and generalizability of the presented work.

## Data Availability

The original contributions presented in the study are included in the article/supplementary material. Further inquiries can be directed to the corresponding authors.
